# Exosomes in Cancer Progression and Therapy Resistance: Molecular Insights and Therapeutic Opportunities

**DOI:** 10.3390/life13102033

**Published:** 2023-10-09

**Authors:** Madita Wandrey, Jadwiga Jablonska, Roland H. Stauber, Désirée Gül

**Affiliations:** 1 Nanobiomedicine/ENT Department, University Medical Center Mainz, Langenbeckstraße 1, 55131 Mainz, Germany; wandrey@uni-mainz.de (M.W.); rstauber@uni-mainz.de (R.H.S.); 2 Translational Oncology/ENT Department, University Hospital Essen, Hufelandstraße 55, 45147 Essen, Germany; jadwiga.jablonska@uk-essen.de; 3 German Cancer Consortium (DKTK) Partner Site Düsseldorf/Essen, 45147 Essen, Germany

**Keywords:** exosomes, cancer, cancer therapy, therapy resistance, resistance transmission

## Abstract

The development of therapy resistance still represents a major hurdle in treating cancers, leading to impaired treatment success and increased patient morbidity. The establishment of minimally invasive liquid biopsies is a promising approach to improving the early diagnosis, as well as therapy monitoring, of solid tumors. Because of their manifold functions in the tumor microenvironment, tumor-associated small extracellular vesicles, referred to as exosomes, have become a subject of intense research. Besides their important roles in cancer progression, metastasis, and the immune response, it has been proposed that exosomes also contribute to the acquisition and transfer of therapy resistance, mainly by delivering functional proteins and RNAs, as well as facilitating the export of active drugs or functioning as extracellular decoys. Extensive research has focused on understanding the molecular mechanisms underlying the occurrence of resistance and translating these into strategies for early detection. With this review, we want to provide an overview of the current knowledge about the (patho-)biology of exosomes, as well as state-of-the-art methods of isolation and analysis. Furthermore, we highlight the role of exosomes in tumorigenesis and cancer treatment, where they can function as therapeutic agents, biomarkers, and/or targets. By focusing on their roles in therapy resistance, we will reveal new paths of exploiting exosomes for cancer diagnosis and treatment.

## 1. Introduction

Exosomes are nanosized extracellular vesicles (EVs) that were first described as early as 1983 [1,2]. While they were considered a cellular mechanism for waste disposal until the 1990s, research has shown that they carry complex cargoes. By delivering their cargo to neighboring and distant cells, exosomes participate in cell–cell communication and influence the phenotype of recipient cells. The respective cargo is highly dependent on the cell of origin [3,4,5]. Exosomal communication contributes to fundamental physiological processes like cell homeostasis, immune response regulation, and senescence. As exosomes are released by almost all cell types, aberrant cells, such as cancer cells, also readily shed exosomes. These exosomes are part of the tumor microenvironment (TME) and play an important role in cancer progression, metastasis, immune response, and therapy. They also contribute to the acquisition and transfer of therapy resistance by promoting epithelial–mesenchymal transition (EMT) and delivering functional proteins and non-coding RNAs (ncRNAs) [6,7]. Therapy resistance is one of the major challenges in the treatment of various cancer entities, as it is the main cause of relapses and poor survival outcomes.

## 2. Exosome Structure and Contents

Exosomes are characterized by their small size (30–200 nm) and endosomal origin, allowing for differentiation from other types of EVs like microvesicles (100–1000 nm) and apoptotic bodies (1–5 µm) [8].

**Figure 1:**
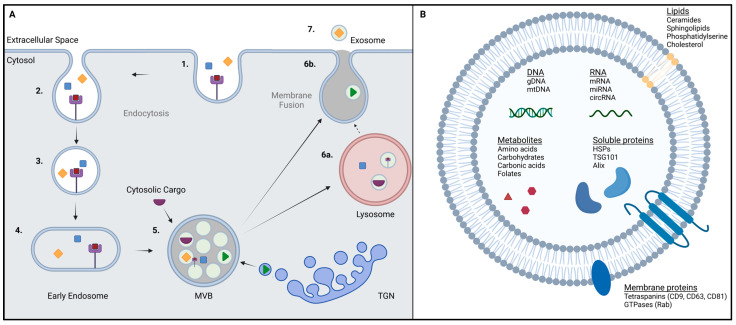
(**A**) Schematic overview of exosome biogenesis via the endosomal pathway. Starting with (receptor-mediated) endocytosis (**1.**,**2.**), intracellular vesicles are formed (**3.**) and converge into early endosomes (**4.**). After maturation to late endosomes, intraluminal vesicles are formed through inward budding of the endosomal membrane, resulting in the MVB (**5.**). Upon fusion of the MVB with lysosomes (**6a.**), contents are typically recycled or degraded. Fusion of the MVB or lysosome with the plasma membrane (**6b.**) leads to the release of exosomes into the extracellular space (**7.**). (**B**) Schematic illustration of a single exosome and its bioactive cargo and components. MVB = Multivesicular body. TGN = Trans-Golgi network. Symbols indicate different endosomal cargoes, such as peptides and proteins, metabolites, and nucleic acids (as depicted in B). Purple squares symbolize ligand for receptor-mediated endocytosis. Created with BioRender.com.

The contents of exosomes are highly complex and include proteins, nucleic acids, metabolites, and lipids (Figure 1B). To date, 41,860 proteins, 7784 RNAs, and 1116 lipids from 286 studies have been entered into the ExoCarta database [9]. Typically, exosomes are enriched in tetraspanins (CD63, CD81, CD9), heat shock proteins (HSP70, HSP90), multivesicular body (MVB)-formation-associated proteins (TSG101, ALG-2-interacting protein X/Alix), and proteins related to membrane transport and fusion (GTPases). These proteins serve as exosome markers, allowing for their distinction from other EVs and even facilitating exosome isolation [10]. Among the RNA species contained in exosomes, microRNAs (miRNAs) are the most abundant in plasma-derived exosomes. Upon their uptake, miRNAs can alter the phenotype of the recipient cell through modifications of its protein expression [11]. As for DNA, it has been shown that more than 90% of cell-free DNA in plasma is associated with exosomes [12]. While DNA of genomic (gDNA) as wells as mitochondrial (mtDNA) origin have been found in exosomes, the packaging mechanisms remain largely unknown. It is thought that the exosomal packaging of DNA is a mechanism of maintaining cell homeostasis but also a means of altering the recipient cell by incorporating the DNA into its genome [13]. Among the metabolites found in exosomes are amino acids and their derivatives, carbohydrates, carbonic acids, and folates [5]. Apart from their cargo, exosomes also have a specific lipid composition. Generally, exosomes are enriched in cholesterol, ceramides, sphingolipids, and phosphatidylserine. Additionally, exosomes can also contain lipolytic enzymes, which can autonomously produce units of bioactive lipids [14,15].

## 3. Exosome Shaping and Signaling

### 3.1. Exosome Biogenesis

Exosomes originate from the endosomal pathway, typically starting with the (receptor-mediated) endocytosis of extracellular materials through the invagination of the plasma membrane (Figure 1A). The resulting vesicles converge into early endosomes. These early endosomes not only contain extracellular components but also plasma membrane proteins [15,16]. Through V-ATPase-mediated acidification, they mature into late endosomes. Late endosomes can also receive cargo-loaded vesicles from the Trans-Golgi network [17]. From the inward budding of the endosomal membrane, small intraluminal vesicles (ILVs) are formed. The formation of ILVs requires the activity of the endosomal sorting complex required for transport (ESCRT), which is initiated by the binding of ubiquitinated proteins to the ESCRT-0 subunit in the endosomal membrane [18,19]. Alternatively, there are also ESCRT-independent routes for ILV formation based on ceramide- or tetraspanin-enriched microdomains within the endosomal membrane [20,21,22]. The ILVs are contained within the MVB, and upon fusion of the MVB with the plasma membrane, they are released into the extracellular space as exosomes [15]. Alternatively, MVBs can fuse with lysosomes, which leads to the degradation of their contents, although there have also been reports of lysosomes fusing with the plasma membrane and releasing exosomes [23]. Other reports also mention exosomes originating directly from the plasma membrane. Budding from endosome-like microdomains, they carry classic exosome markers like CD63 and CD81 [24,25]. However, the amount of evidence is still limited, leaving this route of biogenesis up for debate [8].

### 3.2. Exosome Distribution and Bioactivity

Exosome biodistribution occurs locally and systemically. Exosomes can undergo multiple cycles of uptake and release, allowing for deep tissue penetration [8]. Most studies on exosome biodistribution come from pharmacokinetic research, meaning they use heterologous exosomes. The oral administration of heterologous exosomes results in their distribution to almost all organs, including the liver, lung, kidney, colon, and brain [26,27]. Intravenous administration leads to the fast elimination of heterologous exosomes from the bloodstream, while nasal administration has been shown to direct them to the brain [28,29]. Exosome biodistribution and cell or organ tropism are considerably influenced by many factors, including the cell of origin, the membrane constitution, and the overall patho-/physiological state of the host, as well as dose and route of administration in the case of heterologous exosomes [30]. It is very likely that the biodistribution of autologous exosomes differs significantly from that of heterologous ones. Nevertheless, autologous exosomes have been found in various bodily fluids, including blood, urine, breast milk, saliva, and interstitial fluid [8].

**Figure 2:**
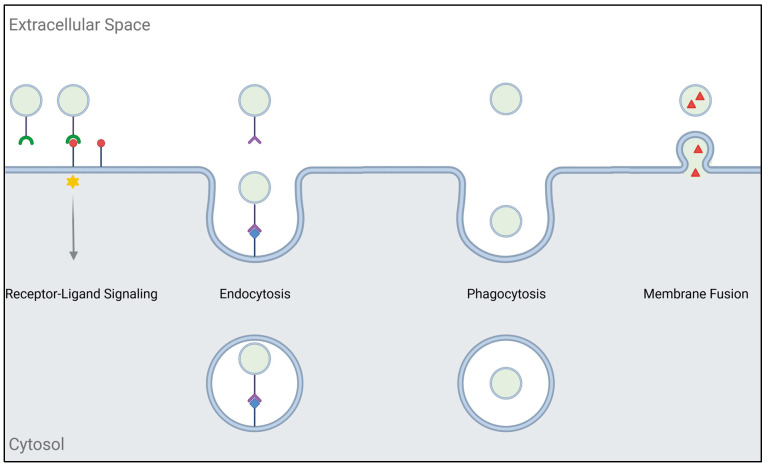
Schematic overview of possible routes for exosome bioactivity. Interaction of exosomal ligands (green) and cellular receptors (red circle) can trigger downstream signaling cascades ((**left**); marked by an asterisk). Alternatively, exosomes can be internalized by endocytosis (receptor-ligand interaction in purple/blue) or phagocytosis (**middle**). Another possibility is the fusion of exosomal and cellular membrane, allowing the direct release of exosomal cargo (red triangle) into the cytoplasm (**right**). Created with BioRender.com.

Upon reaching the recipient cell, there are different possibilities for exosomes to elicit bioactivity (Figure 2). Ligands in the exosomal membrane can bind to cellular receptors, causing downstream signaling cascades. One study has shown that exosomes released by dendritic cells carry tumor necrosis factor (TNF) and TNF-related apoptosis-inducing ligand (TRAIL), which bind to the TNF receptors on tumor cells and activate caspases, eventually resulting in apoptosis [31]. Alternatively, exosomes can be taken up by endocytosis, phagocytosis, or plasma membrane fusion. Fusion with the plasma membrane is most likely Rab- and SNARE-mediated and leads to the direct release of exosomal cargo into the recipient cell [32,33]. However, the primary route for exosome uptake is believed to be internalization by endocytosis or phagocytosis. Typically, endocytosis is receptor-mediated and results in clathrin-coated intracellular vesicles that eventually undergo uncoating and converge into early endosomes. Clathrin-independent endocytosis occurs at lipid rafts, which are membrane microdomains enriched in cholesterol, sphingolipids, and GPI-anchored proteins [16]. Phagocytosis mainly occurs in immune cells like macrophages and dendritic cells. During phagocytosis, the plasma membrane is deformed and engulfs extracellular materials, including bacteria, dead cells, and EVs. The resulting phagosome is internalized and directed to the endosomal pathway [34]. The typical endpoint of the endosomal pathway is the lysosome, where recycling and degradation take place. In order to achieve signaling, exosomes or their cargo have to bypass lysosomal degradation [8]. Some cargoes, for instance, transforming growth factor (TGF) β-1, can be activated by acidification within the endosome [35]. It has also been shown that cargo can passively diffuse into the cytoplasm [36]. Rab5/Rab7-positive endosomal vesicles have been found to interact with the endoplasmic reticulum (ER). Since protein translation occurs at the ER, this would be a favorable route for exosomes carrying mRNA or miRNA [37,38]. The nucleoplasmic reticulum (NR) is a compartment consisting of invaginations penetrating into the nucleoplasm. Upon the interaction of the NR with late endosomes, exosomal cargo could enter the nucleus [39]. Other possible routes for bypassing degradation are retrograde trafficking from the endosome to the Golgi apparatus, the fusion of endosomal and exosomal membranes, and release into the cytoplasm by endosome or lysosome rupture [8].

## 4. Exosome Isolation and Analysis

### 4.1. Isolation of Exosomes

One major challenge in studying exosomes is their efficient and reproducible isolation since they are small in size, low in density, and mixed with various other components if isolated from bodily fluids. Depending on the downstream application, there are different techniques in use, with the most common ones being ultracentrifugation, size-based isolation, polymer precipitation, and immunoaffinity capture. There are also numerous commercial exosome isolation kits available that rely on the aforementioned methods [40,41].

Ultracentrifugation (UC), also referred to as differential centrifugation, remains the gold standard of exosome isolation and is used by around 50% of researchers [41]. UC relies on the differences in size and density between exosomes and other components of the sample [41,42]. Typically, the protocol starts with a series of lower-speed centrifugations to remove live cells, dead cells, and debris (at 300× *g*, 2000× *g*, and 10,000× *g*, respectively). Alternatively, this can be achieved by filtration. Following this are two high-speed centrifugations at 100,000× *g* to precipitate the exosomes [41,43]. The advantages of UC are that it is a well-established method suitable for a variety of sample types and volumes. The downsides of UC are that it is a rather time-consuming method, and due to the high centrifugation speeds, exosomes can be damaged, and their biological functions can be altered. Therefore, UC is more suitable for proteomic or other molecular analyses and less suitable for functional assays. Also, as it is based on size and density, impurities like protein aggregates or organelles can co-precipitate [40,44]. To reduce such impurities, density-gradient UC was introduced, where separation media with different densities are applied. However, this method is rather elaborate and even more time-consuming than classic UC, limiting its application [45,46].

The most common size-based techniques are size-exclusion chromatography (SEC) and ultrafiltration (UF). For SEC, samples are added to columns containing a porous gel (e.g., Sepharose), where larger particles cannot enter the pores and therefore elute faster than the small exosomes trapped in the pores. Since this method works with a low centrifugal force or even gravity, isolated exosomes remain intact and biologically active [47]. However, SEC relies solely on size, which leads to contamination with other particulates in a similar size range. Therefore, SEC is often applied in combination with other methods, like UC [48].

UF uses membranes with different molecular weight cutoffs (MWCOs) to separate exosomes from larger particles. Different forces can be used to drive the sample through the membranes, including centrifugation, pressure, and electric charge. As for SEC, UF suffers from impurities of similar-sized particulates and additionally from low recovery rates due to unspecific binding to or clogging of the membranes [44,49].

Polymer precipitation is a common method for isolating viruses, which share a lot of common features with exosomes [50]. The most commonly used reagent for polymer precipitation is polyethylene glycol (PEG), which reduces the solubility of exosomes, allowing their precipitation at lower centrifugation speeds. While this method is quick and easy, it is also prone to contamination with particles with similar biophysical properties, and it can be difficult to remove the polymer reagent after isolation [40].

Immunoaffinity capture relies on the interaction between antigens and specific antibodies. As described in Section 3.1, exosomes carry a number of general marker proteins that can serve as antigens for capture. The respective antibodies are fixed to a carrier, mostly beads or chromatography matrices. Through the binding of the exosomal antigen to the fixed antibody, exosomes can be separated from contaminants in the sample. Such methods yield high-purity exosomes but are also rather high in cost and time-consuming. Additionally, the isolated exosome population is highly dependent on the choice of antigen(s), thus not reflecting the total exosome population [41,51,52].

Recently, microfluidic devices have emerged as promising tools to combine enrichment and analysis of exosomes. Generally, many of these devices also rely on classic isolation techniques based on physical and biochemical properties. Microfluidic devices based on physical properties contain porous membranes, nanofilters, microvilli, or electrical or acoustic fields to trap and separate exosomes. Biochemical-property-based devices contain mobile or stationary antibody-coated media [41]. There are also devices combining both approaches. Notably, some platforms even offer the possibility of the real-time analysis of the isolated exosomes, for example, by subsequent imaging or biomarker detection [53,54].

The choice of a suitable isolation method is highly dependent on the research focus and downstream application. At the same time, the obtained results may vary depending on the applied isolation method, making this choice pivotal for any exosome research. For more detailed information on methods for exosome isolation and their specific implications, we refer the reader to more thorough reviews on the topic [44,55,56,57].

### 4.2. Analysis of Exosomes

To validate successful exosome isolation and gain further insights into their patho-/biological functions, the downstream analysis of exosomes is crucial. Exosomes are typically analyzed for their physical and/or biochemical properties. Physical properties include particle size and concentration, while biochemical properties focus on the exosomes’ composition and cargo.

Physical properties can be analyzed using dynamic light scattering (DLS), nanoparticle tracking analysis (NTA), tunable resistive pulse sensing (TRPS), and electron microscopy (EM). DLS is an easy-to-use method that allows the analysis of very small sample volumes. However, DLS has a rather low resolution and tends to skew data toward larger particles [58,59]. NTA is a sensitive method that can readily detect particles in the size range of exosomes, but its major downside is the requirement for rather large sample volumes of around 0.5 mL [59,60]. TRPS is a technique that can assess size, concentration, and surface charge at a single-particle level. While TRPS is suitable for small-sized particles, there are many factors that have to be adjusted for the measurement, like the pore size, current, and choice of calibration particles. When it comes to EM, there are different approaches available, with the most common ones in EV research being transmission EM (TEM) and scanning EM (SEM). Comparing the two, TEM reaches higher resolutions but also needs more advanced sample preparation, as the samples have to be very thin (≤1 µm) to allow the passage of electrons [61,62]. Both methods yield satisfying results for the size assessment of exosomes, but the morphological appearance can differ based on the chosen approach and respective sample preparation [63]. In the Minimal information for studies of extracellular vesicles 2018 (MISEV2018) guidelines published by the International Society of EVs, it is advised to use two complementary techniques to assess EVs at a single-particle level. This should include one image-based method, like EM, and one of the other methods for assessing physical properties [64].

For the analysis of biochemical properties, there are two main groups of methods: immunodetection-based methods and proteomics. Common immunodetection methods include Western blotting and flow cytometry. Western blotting is a quite easy-to-use method that is available in many labs. Western blotting technically allows for the detection of surface markers as well as cargoes since exosomes are lysed before analysis [65]. Unfortunately, Western blotting is highly dependent on the used antibodies when it comes to specificity and reproducibility. Additionally, it only allows for the assessment of a very limited number of proteins in one sample [59]. Flow cytometry requires a single-particle laminar flow of antibody-labeled particles. To achieve this with EVs can be difficult because they are prone to aggregation. As for exosomes, they are also too small to be detected by standard flow cytometers, which detect in a size range of 1–15 µm [66]. Therefore, they are typically bound to beads by either an antibody–antigen interaction or covalent conjugation [67]. Alternatively, the vesicle flow cytometry (VFC) approach is based on staining the vesicle membranes for size and concentration measurements. Antibody staining can give additional information about surface protein expression. To avoid misinterpretation based on aggregation, the measurement of serial dilutions of the sample is advised [68]. Whether VFC can be used for exosomes highly depends on the flow cytometer’s detection limit. Flow cytometry generally allows for high-throughput analysis of exosomes and can identify different expression patterns within one sample. However, as for Western blotting, only a limited number of markers can be assessed in one sample, and the outcome might vary depending on the used antibody. As the particles remain intact, analysis is limited to surface markers. Proteomic analysis of exosomes mainly relies on mass spectrometry (MS). Beyond proteomic research, MS is also a suitable method for the analysis of lipids and metabolites, but so far, proteins remain the main focus of MS-driven exosome research [69]. The MISEV2018 guidelines suggest showing at least three positive and one negative marker for the respective EV type on a protein level using any of the aforementioned methods.

Since the 2018 update, the MISEV guidelines also recommend describing EVs quantitatively. One option would be the particle count, as analyzed by DLS, NTA, or TRPS. Other options include total protein content and total lipid content [64]. As mentioned before, MS can quantify proteins and lipids. However, it is a rather advanced and expensive method, which is hardly used for routine isolation verification. Standard procedures for protein content measurements are colorimetric assays, for example, the Bradford assay. The readout of the Bradford assay is based on a comparison to a reference curve. Accordingly, the Bradford assay can only detect proteins within the linear range of its reference curve. Highly purified or low-concentrated EV preparations might fall outside of this range, leading to an underestimation of the protein content. Conversely, overestimation of the protein content can result from the co-precipitation of protein contaminants like albumin [64]. Other options for colorimetric protein assays include the Pierce BCA assay and the Lowry assay, which both rely on the biuret reaction and the subsequent colorimetric detection of cuprous cations. Lipid content can be measured using the colorimetric sulfo-phospho-vanillin (SPV) assay [70,71]. As for the Bradford assay, the SPV assay uses a reference curve, and lipid concentrations can only be reliably calculated within its linear range; otherwise, lipid content can be over- or underestimated. It is also not established whether lipid assays like the SPV assay can detect all EVs with the same efficacy, independent of their specific lipid composition [64]. Therefore, a conclusive interpretation of protein and lipid content data is nearly impossible, as both require stable input concentrations within the assay’s linear range and differ significantly based on the exosome source and isolation method. Unfortunately, the lack of details regarding exosome production and isolation in many publications further contributes to this issue, highlighting the need for standardized procedures.

## 5. Exosomes in Cancer Development and Progression

Cancer progression is a multi-step process, ultimately resulting in the development of malignancy. Being part of the tumor microenvironment, exosomes have been suggested to regulate multiple different processes of tumorigenesis, such as angiogenesis, metastasis, and immune response.

### 5.1. Angiogenesis

As cancer cells have a high demand for oxygen and nutrients, angiogenesis is crucial for cancer progression. Angiogenesis is induced by an imbalance between anti- and pro-angiogenic factors, which is favored by hypoxic conditions [72]. Interestingly, exosome release has been found to be elevated in cells exposed to hypoxia [73]. Under hypoxic conditions, cancer cells release exosomes enriched in miR-23a, which promotes angiogenesis by targeting HIF-1α, ZO-1, and prolyl hydroxylase [74,75]. Exosomes also deliver pro-angiogenic miRNAs like miR-9, miR-92a-3p, miR-205, and miR-135b, which are involved in endothelial cell migration and tube formation [76,77,78,79]. Melanoma- and glioblastoma-cell-derived exosomes have been shown to carry pro-angiogenic factors like VEGF and IL-6 [80,81]. Exosomes from ovarian cancer cells carry increased levels of soluble E-Cadherin, which activates angiogenic signaling in endothelial cells [82]. Exosomes from nasopharyngeal cancer and myeloma are enriched in matrix metalloproteinases (MMPs) like MMP-9 and MMP-13, which are also involved in angiogenesis induction [83,84]. In head and neck squamous cell carcinoma (HNSSC), small EVs from patients with advanced-stage disease stimulated significantly increased tubulogenesis, migration, and proliferation of endothelial cells compared to those from healthy donors [85]. Upon treatment with exosomes derived from leukemia cells, endothelial cells show increased expression of vascular cell adhesion molecule 1 (VCAM-1) and intercellular adhesion molecule 1 (ICAM-1), which have been associated with neovascularization [86]. The exosomal delivery of surface tetraspanin Tspan8 has also been suggested to promote angiogenesis in colorectal cancer and adenocarcinoma [87].

In summary, exosomes play a significant role in angiogenesis by delivering pro-angiogenic factors and miRNAs, promoting endothelial cell migration, tube formation, and neovascularization in various cancer types.

### 5.2. Metastasis

Another major step in cancer progression is metastasis, which is responsible for 90% of tumor-related deaths [88]. In order to metastasize, tumor cells need to detach from the original tumor mass and gain migratory potential. This process involves extracellular matrix (ECM) degradation and epithelial–mesenchymal transition (EMT). As mentioned before, tumor-derived exosomes (TDEs) can carry MMPs, which mediate ECM degradation. Additionally, TDEs have been shown to carry miRNAs that promote MMP expression. For example, melanoma-derived exosomes can enhance MMP-2 and MMP-9 expression in fibroblasts through the delivery of miR-21 [89]. In a triple-negative breast cancer (TNBC) model, cancer cells released exosomes enriched in MMP-1. The ingestion of these exosomes by different TNBC cell lines leads to increased MMP-1 secretion, favoring EMT [90]. EMT is a cellular process that leads to a loss of epithelial and a gain of mesenchymal characteristics. During EMT, cells lose their apical–basal polarity, as well as cell–cell and cell–ECM adhesions [91]. There are multiple known signaling pathways that can induce EMT through EMT-promoting transcription factors (EMT-TFs). Prominent examples of EMT-TF families are SNAIL, SLUG, TWIST, and ZEB [92]. A very extensively studied EMT signaling pathway in tumor research is the Wnt/β-catenin pathway. Several Wnt ligands are delivered by TDEs, including Wnt1, Wnt4, and Wnt5a [80,93,94,95]. Exosomal Wnt10b delivered by fibroblast-derived exosomes induces EMT in breast cancer cells [96]. Exosomal Tenascin-C (TNC), an extracellular matrix protein, has also been shown to induce EMT in breast cancer cells through the Wnt/β-catenin signaling pathway [97]. Exosomes derived from cancer-associated fibroblasts deliver miR-34a-5p, resulting in β-catenin/SNAIL-mediated EMT induction in oral squamous cell carcinoma (OSCC) [98]. The delivery of circABCC1 by colorectal-cancer-cell-derived exosomes also induces EMT through Wnt/ß-catenin signaling [99].

In OSCC, exosomal miR-21 induces EMT, while miR-21 inhibition leads to reversed EMT and cancer stem cell (CSC) phenotype [100]. In bladder cancer, increased levels of exosomal casein kinase II α and annexin A2 were associated with EMT [101]. Another study showed that the treatment of epithelial tumor cells with exosomes from head and neck SCC patients’ plasma could induce the expression of mesenchymal markers like Vimentin, N-Cadherin, SNAIL, TWIST, SLUG, and ZEB-1. This effect was lost if exosomes were isolated after patients underwent photodynamic therapy [102]. There are also reports suggesting the involvement of TDEs in pre-metastatic niche formation. Specific integrin patterns and adhesion molecules on the TDEs’ surfaces lead to targeted delivery to specific cell types or organs and promote pre-metastatic niche formation [103,104]. In gastric cancer, exosomal delivery of EGFR to liver stromal cells has been associated with the upregulation of hepatocyte growth factor, which represents a binding partner for c-MET and thereby facilitates the landing of metastatic cancer cells [105]. Melanoma-derived exosomes have been shown to prime lymph nodes for metastasis by upregulating proteins involved in melanoma cell recruitment, ECM modification, and angiogenic growth factors [106].

Collectively, a substantial amount of evidence supports the role of exosomes in ECM degradation and EMT, which in turn favors metastasis.

### 5.3. Cancer Immunity

The immune response against malignant cells is of critical importance for the progression and invasion of the tumor. TDEs have been suggested to take part in both anti- and pro-tumor immune reactions.

The anti-tumor response is mainly based on antigen presentation on the TDEs’ surfaces [107]. For example, the presentation of HSP70 on exosomes induces CD8^+^ T-cells and activates NK cells and macrophages [108,109,110]. Exosomes from Rab27a-overexpressing lung cancer cells are able to promote CD4^+^ T-cell proliferation through the activation of MHC-II, CD80, and CD86 on dendritic cells (DCs) [111]. When breast cancer cells are treated with the DNA-double-strand-break-inducing agent Topotecan, they release exosomes that reinforce anti-tumor immunity by activating DCs and CD8^+^ T-cells [112].

However, the majority of TDEs mediate immune-inhibitory functions. It has been shown that exosomes from head and neck cancer (HNC) patients carry immunosuppressive protein cargoes and mediate suppressive effector T-cell functions [113]. For example, cancer-derived exosomes carry TGFβ and PD-L1 [114,115,116,117]. PD-L1 is an immunosuppressive ligand strongly associated with immune evasion in cancer [118]. In agreement, PD-L1-carrying small EVs block the activity of NK cells and T-cells and support apoptosis in CD8^+^ T-cells in HNSCC [117]. Elevated levels of exosomal PD-L1 have been associated with poor patient prognosis in different cancer entities [119].

Another important immunosuppressive axis in cancer is the Fas/FasL pathway. It is considered crucial for cancer-mediated immunosuppression and T-cell apoptosis [120]. In agreement, exosomal presentation of FasL and galectin-9 has been associated with apoptosis induction in activated T-cells [121,122]. Importantly, small-EV-associated FasL levels are reduced in advanced HNSCCs, while Fas is increased for up to six months after therapy [117].

The downregulation of MHC I plays an important role in cancer immunosurveillance. It has been described in 40–90% of human tumors, often correlating with a worse prognosis [123]. Low levels of MHC-I on glioma-cell-derived exosomes are associated with the dysfunction of CD8^+^ T-cells, while the upregulation of exosomal MHC-I restored their anti-tumor response [124].

Myeloid cells, such as macrophages, neutrophils, and DCs, strongly influence anti-cancer immune responses. TDEs can inhibit the differentiation of DCs and the function of their cytokines by delivering miR-203. EVs have also been shown to stimulate the pro-tumor activity of neutrophils and to increase their chemotaxis in melanoma [125]. Moreover, there are numerous reports on exosome-driven stimulation of macrophage polarization toward the immunosuppressive and thus cancer-promoting M2 phase [107]. For example, exosomes from SNAIL-expressing HNC cells have been found to be enriched in miR-21. Upon the uptake of the miR-21-containing exosomes by CD14^+^ monocytes, the expression of M1 markers was suppressed, while that of M2 markers increased [126]. Exosomal delivery of tyrosine kinase with immunoglobulin and epidermal growth factor homology domains 2 (TIE2) from cervical cancer cells to macrophages leads to an M2 phenotype and promotes angiogenesis [127]. TDEs can even influence macrophage polarization in the pre-metastatic niche through TLR2 and NF-κB signaling [128]. Thus, TDEs play an important role in the regulation of anti-tumor immune responses, providing an important target that could be addressed therapeutically in order to impair tumor progression.

By delivering suppressive signals and proteins to immune cells, exosomes substantially contribute to an immunosuppressive microenvironment, ultimately resulting in tumor immune evasion.

## 6. Exosomes in Cancer Therapy and Treatment

Given the compelling evidence supporting the critical involvement of exosomes in the development and progression of cancer, translating this knowledge into clinical use is a current research interest. Seeing as exosomes can deliver their cargo to specific cells, exploiting them as vehicles for targeted drug or signal delivery is a promising approach. Moreover, TDEs carry distinct markers, allowing their use as biomarkers for cancer detection and therapy monitoring. An alternative treatment approach is to target TDEs to prevent disease progression.

### 6.1. Exosomes as Drug Carriers and Therapeutic Agents

The targeted delivery of drugs to cancer cells is one of the major challenges in cancer therapy research. A widely studied approach is the use of nanoparticles (NPs) as drug carriers, offering sustained drug release, prolonged bioavailability, an enhanced permeation and retention (EPR) effect on tumors, and few side effects. For example, in a cell spheroid model of HNSCC, the delivery of cisplatin (CDDP) by NPs was able to overcome CDDP resistance and successfully eradicate cancer cells [129]. Copper(II) bis(diethyldithiocarbamate) (CuET) NPs successfully induced copper-dependent programmed cell death (cuproptosis) in non-small lung cancer cells and showed potent anti-tumor effects in a CDDP-resistant tumor model in vivo [130]. However, the binding of bodily proteins and biomolecules to the NPs, the so-called NP-corona, often limits the desired effects in vivo [131,132,133]. Exosomes can be viewed as naturally occurring NPs, which have a high bioavailability and few side effects, therefore representing a promising carrier for therapeutic drugs. Loading the respective drugs into exosomes can be achieved by modifying the donor cells to directly incorporate the drug into the exosomes. If this is not possible, purified exosomes can be loaded in vitro using electroporation, sonification, or passive diffusion [134,135]. The delivery of paclitaxel (PTX) via exosomes was shown to be more efficient than free-drug or liposome delivery in a multi-drug-resistant cancer cell model [135]. In mice, coating exosomes with different chemotherapeutics leads to a complete loss of side effects compared to free drugs while effectively inhibiting tumor growth [47]. Chemical or genetic modifications of the exosomal surface also allow for a more targeted delivery of their cargo. The conjugation of ApoA-1 mimetic peptides to lipids allows for the targeted delivery of Methotrexate-loaded exosomes to primary glioma cells [136]. Exosomes engineered to carry a peptide that readily binds to c-Met were successfully targeted to c-Met-overexpressing triple-negative breast cancer cells in vitro and in vivo, leading to increased cellular uptake and tumor apoptosis [137]. Another option for targeting exosomes is the use of antibody mimetics like affibodies. Affibodies are based on the immunoglobulin binding domain of protein A and can bind target proteins with high specificity. Surface Her2-affibody-expressing exosomes from genetically engineered HEK293T donor cells successfully deliver PTX and miR-21 to Her2-expressing colorectal cancer cells and show anti-tumor effects in mice [138]. Genetically engineered exosomes expressing a fragment of IL-3 on their surfaces effectively target IL3-receptor-overexpressing chronic myeloid leukemia cells and inhibit cell growth by delivering Imatinib or BCR-ABL siRNA [139].

Exosomes can also be used as therapeutic agents to induce immunogenic cell death (ICD). Exosomes derived from DCs are known to carry classic antigen-presenting molecules like MHC-I/II and CD1a-d, allowing them to activate the anti-tumor immune response [140]. For example, DC-derived exosomes are able to prime antigen-specific CD4 and CD8 T-cells through MHC-I/II expression and antigen presentation. There are multiple ongoing clinical trials exploring the effectivity of DC exosomes as cancer vaccines [141]. In breast cancer, synthetic multivalent antibody retargeted exosomes (SMART-Exo) have been exploited to activate and redirect T-cells to EGFR- or HER-2-expressing cancer cells by displaying the respective antibodies on their surface [142,143]. CD40-ligand-expressing exosomes from modified lung cancer cells are able to activate DCs in vitro and enhance the anti-tumor activity of T-cells in a mouse model [144]. Engineered A-lactalbumin exosomes loaded with the ICD inducers human neutrophil elastase (ELANE) and Hiltonol specifically induce ICD in breast cancer cells, which in turn leads to the activation of DCs and CD8^+^ T-cells. ICD can also be triggered by the exosomal delivery of galectin-9 siRNA and the oxaliplatin prodrug in pancreatic ductal adenocarcinoma [145]. In some cases, inflammation can also contribute to tumor growth [146]. Exosomes have also been proposed as decoys for the proinflammatory cytokine TNFα by displaying the TNFα-binding domain of human TNF receptor-1 on their surface [147].

Exploiting exosomes as therapeutic agents is a promising approach, even beyond cancer treatment. However, most studies are still pre-clinical, and further efforts will be necessary to translate the current knowledge into clinical application.

### 6.2. Exosomes as Biomarkers

Recently, liquid biopsies have emerged as a minimally invasive procedure that can complement the management of cancer patients during standard therapy. Compared to tissue biopsies, liquid biopsies are lower in cost and easily performed, enabling multiple samplings during a patient’s treatment [148]. Among the examined biomarkers are circulating tumor cells (CTCs), cell-free DNA, circulating RNA, and exosomes [149,150]. Exosomes are more abundant and stable in the circulation than CTCs and nucleic acids, making them promising candidates for biomarkers [151]. In breast cancer, a correlation between the total amount of exosomal miRNA and malignancy and prognosis could be established [152]. LncRNA H19 from serum exosomes is significantly elevated in breast cancer patients compared to healthy donors, making it a promising diagnostic marker [153,154]. Exosomal miR-106b is elevated in lung cancer patients compared to healthy individuals and could be linked to cancer cell migration and lymph node metastasis [155]. Upregulation of miR-21 and miR-4257 in exosomes was associated with disease recurrence in non-small-cell lung cancer [156]. In HNSCC, exosome levels are elevated in advanced-stage tumors, with exosome amounts nearly doubling with disease progression [113,157]. One study showed that a high expression of the mesenchymal markers N-cadherin and TGF-β1 on plasma-derived exosomes of HNSCC patients is associated with increased tumor proliferation, migration, and invasion. After photodynamic therapy, they observed a shift to epithelial markers [102]. In a study surveilling HNSCC patients receiving a combination treatment with cetuximab, ipilimumab, and radiation, patients with recurrent disease showed increased levels of total exosome proteins, total exosome ratio, and total CD3^+^, CD3^−^PD-L1^+^, and CD3^+^15s^+^ exosomes compared to patients who remained disease-free [158]. Overexpression of EGFR is a common occurrence in HNSCC, which is also reflected in exosomes [150]. Others have shown that exosomal EGFR and pEGFR are reduced in HNSCC patients after cetuximab treatment, allowing for treatment monitoring [159]. In laryngeal SCC, serum exosomal miR-21 and homeobox transcript antisense RNA (HOTAIR) could be associated with disease progression. Patients with lymph node metastasis showed increased levels of exosomal miR-21 and HOTAIR [160]. In patients with stage III colorectal cancer, levels of exosomal 20S proteasome and MMP9^+^ subpopulations were associated with unfavorable prognostic factors for overall survival [161]. In colon cancer, the lipid profiling of exosomes revealed the molecular species PE 34:2, PE 36:2, pPE 16:0/20:4, and Cer d18:1/24:1 as possible markers for metastasis [162].

Exosomes represent a promising diagnostic and prognostic tool for a variety of different cancer entities and thus have the potential to change the management of cancer patients in the future. However, the lack of standardized isolation methods is hindering the integration of exosomal biomarkers into the clinical routine [163].

### 6.3. Exosomes as Therapy Targets

Since exosomes have been shown to play a role in multiple steps of cancer progression, they are also interesting targets for therapy. GW4869 is a potent inhibitor of the membrane neutral sphingomyelinase (nSMase), an enzyme that hydrolyzes sphingomyelins and thereby generates ceramides. SMase is found in different cellular compartments, including the Golgi apparatus, endosomes, and the plasma membrane. GW4869-induced Smase inhibition has been shown to also potently inhibit exosome release [164]. GW4869-mediated inhibition of exosome release from cancer-associated fibroblasts can reduce the chemoresistance transfer and thus the survival of pancreatic cancer cells. The combination treatment of gemcitabine and GW4869 also led to reduced tumor growth in a mouse model [165]. Treatment with a CD44-targeting nanounit composed of GW4869 and the ferroptosis inducer FE^3+^ induced an anti-tumor immune response to melanoma cells in mice, while no cytotoxic effects of GW4869 could be observed in vitro [166]. Transfection of pancreatic cancer cells with siRAB27B can also reduce exosome release, resulting in a significant decrease in miR-155-induced gemcitabine resistance [167]. Targeting RAB27A with siRNA led to reduced tumor growth and metastasis in mouse models [168,169]. The blood-pressure-lowering drug dimethyl amiloride has been shown to reduce endocytic recycling and, in turn, also exosome release. Treatment with amiloride can reduce tumor growth in mice by preventing the exosomal delivery of HSP72 to myeloid-derived suppressor cells and increase cyclophosphamide-based chemotherapy efficacy [170]. Inhibition of exosome uptake is another possible route to target exosomes for therapy. Enzymatic depletion of cell surface HSPG has been shown to effectively attenuate exosome uptake [171]. Annexin V binds to and blocks surface phosphatidylserine, which is important for membrane adhesion and exosome uptake. Treatment with annexin V was shown to reduce the growth rate and metastatic potential of human glioma xenografts in mice [172].

While there is evidence supporting the exploitation of inhibiting exosome release and uptake as a novel therapeutic approach, further in vivo studies will be necessary to explore the systemic effects of such inhibitors. Assuming that interference with Smase activity and phosphatidylserine adhesion is likely to disrupt other cellular processes, achieving the precisely targeted delivery of inhibitors will be crucial.

## 7. Exosomes in Transmitting Therapy Resistance

The choice of a suitable treatment regimen is highly dependent on the cancer entity, progression stage, and metastasis stage of the tumor. Generally, treatment options include surgical resection, radiotherapy, and systemic therapy, with the ladder including chemotherapy and immunotherapy. Different approaches are often combined to achieve the best possible outcome for the patient. While surgical removal remains a standard procedure for many solid tumors, it is not always an option, depending on the affected anatomical site, and there are also reports suggesting that surgery-induced trauma increases metastasis and recurrence [173]. Non-surgical treatment options are mainly focused on triggering programmed cell death (PD) in cancer cells by inducing cellular stress and DNA damage or enhancing immune surveillance mechanisms [174]. Unfortunately, primary or acquired therapy resistance is a common occurrence, contributing to the nearly 10 million cancer deaths worldwide in 2020 [175]. As intercellular messengers of the TME, exosomes have been suggested to contribute to the development and transfer of therapy resistance in cancer.

### 7.1. Chemotherapy

**Figure 3:**
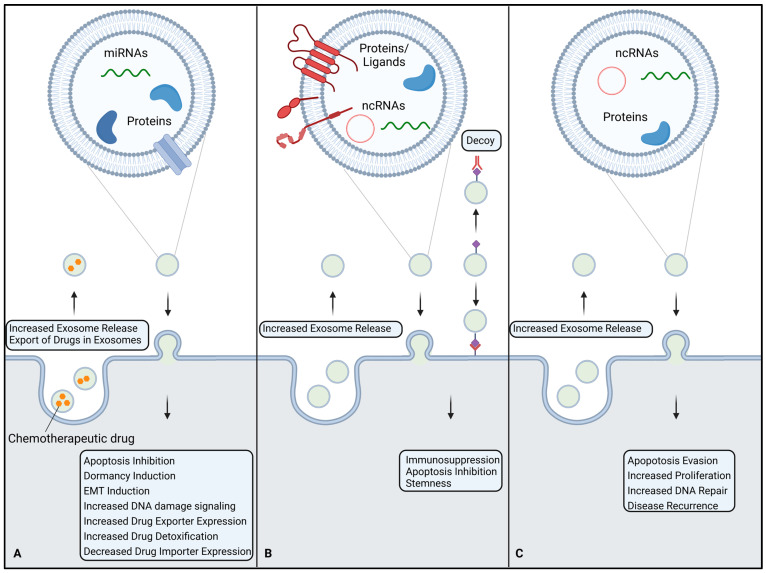
Schematic overview of exosome-related mechanism contributing to therapy resistance. (**A**) Increased release of exosomes and export of active drug within exosomes facilitates chemotherapy resistance. Resistant cells can transfer their resistant phenotype to naïve cells via exosomal delivery of proteins and miRNAs. Upon uptake, this cargo can induce different cellular responses related to chemotherapy resistance. (**B**) Immunotherapy-resistant cells also show an increased release of exosomes. These exosomes can function as decoys for immunotherapeutic drugs or facilitate receptor–ligand signaling that increases therapy resistance. Uptake of exosomes shed by resistant cells can also induce immunotherapy resistance by delivering ncRNAs and proteins. (**C**) Radiotherapy-resistant cells also show an increased amount of exosome release. These exosomes contain ncRNAs and proteins that trigger resistance mechanisms if taken up by naïve cells. Created with BioRender.com.

The primary goal of chemotherapy is to inhibit tumor cell proliferation and, in turn, prevent invasion and metastasis. Unfortunately, resistance to chemotherapeutic agents is common, accounting for 90% of deaths in patients with metastatic tumors [176]. There are two main routes by which cells achieve chemoresistance: a reduction in the intracellular active drug concentration and the evasion of apoptosis. The intracellular active drug concentration is influenced by drug influx, which depends on transporter expression and membrane constitution, as well as active drug export and intracellular drug detoxification. The evasion of apoptosis is achieved by upregulating pro-survival genes and increasing DNA damage repair activity [177]. Exosomes can contribute to the transmission of chemoresistance mechanisms by delivering functional proteins and miRNAs to neighboring cells and contributing to active drug export from their cell of origin (Figure 3A).

Generally, exosome biogenesis is accelerated by treatment with chemotherapeutic drugs [178,179]. There is evidence that drug export via exosomes contributes to resistance in different cancer entities. In ovarian cancer, exosomes from CDDP-resistant cells were shown to incorporate two-fold more CDDP than those from sensitive cells [180]. In adriamycin (ADM)-resistant breast cancer cells, ADM did not accumulate in the nuclei as expected but instead was exported in exosomes [181]. A similar mechanism was observed in gemcitabine-resistant pancreatic cancer cells [182].

Exosomes can also facilitate the horizontal transfer of proteins. Functional drug exporters like P-gp, multi-drug-resistance-associated proteins (MRPs), and ABC transporters ABCA-3 and ABCG-2 have been found in exosomes [7]. Exosomal delivery of P-gp was shown to transfer a chemoresistant phenotype in breast cancer cells [183,184]. Notably, P-gp functionality could be detected as early as two hours after transfer [185]. Docetaxel-resistant prostate-cancer-cell-derived exosomes show elevated levels of P-gp compared to those from sensitive cells. Likewise, exosomes isolated from patients with docetaxel-resistant pancreatic cancer showed higher P-gp levels than those from therapy-naïve patients [186]. Exosomal transfer of MRP-1 and ABCA-3 could confer a chemoresistant phenotype to leukemia cells [187,188]. Exosomes from CDDP-resistant ovarian cancer cells have been shown to carry elevated levels of the drug exporters MRP2, ATP7A, and ATP7B [180]. Mesenchymal stem cell (MSC)-derived exosomes could be shown to prevent 5-fluorouracil (5-FU)-induced apoptosis by activating the CaM-Ks/Raf/MEK/ERK pathway and stimulating the expression of MRPs in a gastric cancer mouse model [189].

Exosomes have also been shown to carry pro-survival proteins like survivin and HSPs. Treatment of hepatocellular carcinoma cells with CDDP or Carboplatin increased the exosomal export of HSPs 60, 70, and 90 [179]. High exosomal survivin levels could be associated with relapse after chemotherapy in prostate cancer patients [190]. PTX-resistant breast-cancer-cell-derived exosomes have also been found to contain increased levels of survivin, leading to apoptosis evasion and resistance transfer [191]. Exosomes isolated from hepatitis-B-associated liver cancer cells increased the expression of Lamp2a and, in turn, increased apoptosis evasion and oxaliplatin resistance in naïve liver cancer cells. However, the details of the underlying mechanism remain unclear [192]. Exosomal transfer of HSP90 from PTX-resistant breast cancer cells to sensitive cells increases their PTX resistance through the degradation of p53. HSP90 was also found to be upregulated in plasma exosomes from patients showing PTX resistance [193].

Among other proteins that are transferred via exosomes is the microsomal triglyceride transfer protein (MTTP), which inhibits ferroptosis and induces resistance to oxaliplatin in colorectal cancer. MTTP levels were found to be increased in the plasma exosomes of colorectal cancer patients with a high body fat ratio, correlating with a poor therapy response [194]. In an esophageal SCC cell model, exosomes from PTX-resistant cells showed higher levels of PD 1 ligand 1 (PDL-1) than those from sensitive cells. Exosomal PDL-1 could transfer the resistant phenotype, putatively by regulating the STAT3/miR-21/PTEN/Akt axis [195]. In an ADM-resistant breast cancer cell model, exosomes transferred resistance via UCH-L1. High levels of UCH-L1 in patient exosomes before therapy onset could be associated with a poor prognosis [181]. Exosomal delivery of CD44 could transfer doxorubicin resistance in a breast cancer cell model. Likewise, exosomal CD44 in the serum of non-responders was significantly higher than in chemotherapy-responsive patients [196]. Exosomes from nasopharyngeal cancer cells treated with CDDP show increased levels of ER-resident protein 44 (ERp44), which enhances the resistant phenotype in recipient cells. The incorporation of ERp44 in exosomes is likely caused by CDDP-induced ER stress [197].

Another mode of resistance transfer via exosomes is the delivery of miRNAs. MiR-21 is one of the most studied oncogenic miRNAs and has been found to play a role in various cancer entities, including glioma, liver cancer, colorectal cancer, prostate cancer, breast cancer, ovarian cancer, and bladder cancer [198]. In oral SCC, exosomal miR-21 was indicated in CDDP resistance transfer by targeting DNA damage signaling [199]. The level of miR-21 is significantly increased in exosomes derived from CDDP-resistant esophageal cancer cells compared to those from sensitive esophageal cancer cells or healthy esophageal cells. Exosomes were also able to transfer the resistant phenotype through miR-21-mediated downregulation of PDCD4 [200]. CDDP-resistant liver cells release exosomes with increased levels of miR-643, which has been shown to transfer resistance to recipient cells by targeting APOL6 and inhibiting apoptosis [201]. In breast cancer cells, Tamoxifen resistance could be transferred to sensitive cells via exosomal miR-221/222 delivery [202]. Exosomal miR-222/223 was shown to induce cancer cell dormancy and subsequent Carboplatin resistance in breast cancer in vitro and in vivo [203]. In another breast cancer model, exosomal miR-451 and miR-326 transferred a P-gp-overexpressing phenotype from donor to recipient cells [204]. When analyzing breast cancer patient exosomes from serum before and after chemotherapy with doxorubicin and paclitaxel, miR-378a-3p and miR-378d could be correlated with therapy resistance. In vitro experiments further revealed that exosomal transfer of miR-378a-3p and miR-378d induced chemoresistance [205]. Exosomes derived from doxorubicin-resistant gastric cancer cells are enriched in miR-501, which can transfer the resistant phenotype to sensitive cells [206]. MiR-196a-3p, which is thought to target the gemcitabine importer hENT1, is upregulated in the exosomes of pancreatic cancer cell lines and in the serum exosomes of patients [207]. Gemcitabine treatment of pancreatic cancer cells can lead to increased amounts of exosomal miR-155, which can induce resistance in recipient cells [167]. Exosomal miR-155-5p has also been associated with EMT induction and the transfer of paclitaxel resistance in gastric cancer cells [208]. Prostate cancer tissue treated with CDDP, doxorubicin, and docetaxel shows increased levels of exosomal miR-27a, which induces chemoresistance by targeting p53. In vitro experiments were able to show that primary prostate fibroblasts co-cultured with cancer cells are a vital source of miR-27a-carrying exosomes [209]. Some miRNAs are also reduced in exosomes upon chemotherapeutic treatment. The miR-30a level is decreased in CDDP-resistant oral SCC cells. Accordingly, exosomal miR-30a was found to be significantly reduced in the serum of oral SCC patients after CDDP treatment, especially those showing recurrent disease [210]. In non-small-cell lung cancer, a reduction in exosomal miR-1273a was found to induce CDDP resistance by increasing Syndecan-binding protein (SDCBP) levels in recipient cells. Patients receiving platinum-based therapy showing decreased plasma exosomal miR-1273a and increased plasma SDCBP levels experienced worse therapeutic outcomes [211]. Research into exosomal miRNAs and other ncRNAs is a growing field, with novel oncogenic and chemoresistance-related candidates emerging continuously.

While there is a lot of evidence for a pivotal role of exosomes in chemoresistance transfer from in vitro experiments, only a limited number of studies providing in vivo data are available. Conditions in the TME differ significantly from those in vitro, potentially leading to divergent kinetics of exosomal resistance transfer in vivo [7].

Interestingly, some studies also suggest exosomes as enhancers of chemosensitivity. Mesenchymal-stem-cell-derived (MSC) exosomes have been implicated in reversing resistance to taxanes, such as docetaxel, in ovarian cancer by delivering miR-146a [212]. Exosomal miR-451a can inhibit EMT in hepatocellular carcinoma cells, resulting in increased PTX sensitivity [213]. Adipose MSCs release exosomes carrying miR-1236, which increases CDDP sensitivity through SLC9A1 downregulation in breast cancer cells [214].

### 7.2. Immunotherapy and Targeted Therapy

Ideally, the cells of the immune system recognize and eliminate aberrant cells like cancer cells, thus preventing tumor growth. Unfortunately, cancer cells have evolved different immune-evasive and -suppressive mechanisms to bypass these natural defenses. The aim of immunotherapy is to stimulate the immune response to eradicate cancer cells more effectively. There are different approaches available, including immune checkpoint inhibitors (ICI), cancer vaccines, or adoptive cell transfer. Given their role in cancer immunity, exosomes also contribute to immunotherapy resistance (Figure 3B).

Treatment with immunotherapeutic drugs can increase the number of secreted exosomes and alter their molecular profile [215]. For example, treatment of colorectal cancer cells with cetuximab leads to the release of exosomes with an increased abundance of miRNAs and proteins activating proliferation and inflammation, while those related to immune suppression are reduced [216].

The only FDA-approved ICIs target PD-1, PD-L1, or cytotoxic T-lymphocyte-associated antigen-4 (CTLA-4), all of which are negative regulators of T-cell immune function. CTLA-4 competes with the co-stimulatory CD28 for its ligands CD80 and CD86. Exosomal miR-424 from colorectal cancer cells can inhibit the CD28-CD80/86 pathway, leading to ICI resistance [217]. PD-L1 expression has been found in and on TDEs from different entities [218]. In metastatic melanoma patients, exosomes were found to carry increased levels of PD-L1, contributing to T-cell suppression. The study suggests that high levels of exosomal PD-L1 reflect T-cell exhaustion, rendering anti-PD-1 treatment with Pembrolizumab futile [116]. Exosomal PD-L1 expression can also circumvent anti-PD-L1 treatment. Exosomal PD-L1 can bind to PD-1 on T-cells regardless of PD-L1 inhibition on the tumor cells. Alternatively, PD-L1-expressing exosomes can bind to the respective inhibitor and thus function as a decoy. Both mechanisms are possible explanations for the continuing immunosuppression despite ICI treatment [219].

Currently, there are three therapeutic cancer vaccines and seven adoptive cell transfer treatments approved by the FDA, although the distinction between the two categories is somewhat blurry [220,221]. So far, there is little knowledge about the role of exosomes in these innovative treatment approaches. For example, one study found that EVs expressing CD19 can be used to boost the expansion and efficacy of chimeric-antigen-receptor-modified (CAR) T-cells in vitro and in vivo [222].

Targeted therapy is mostly based on small molecules and monoclonal antibodies. Monoclonal antibodies can block vital cellular processes or help immune cells to recognize and subsequently eliminate cancer cells, leaving them at the intersection of immunotherapy. They are designed to target antigens that are specific to cancer cells. HER-2-positive exosomes from cell culture and breast cancer patients have been shown to express active full-length HER-2 and bind to Trastuzumab, inhibiting its activity [223]. A similar mechanism was observed in B-cell lymphoma, where CD20-expressing exosomes function as a decoy for Rituximab [224,225]. In a colon cancer model, exosomes could transfer cetuximab resistance to sensitive cells by downregulating PTEN and increasing p-Akt levels [226]. Another study found that exosomes released from MDR colorectal cancer cells increased PD-L1 and Sox2 expression in recipient cells via PI3K/AKT activation and led to the expression of stem-cell-associated markers, resulting in cetuximab resistance [227]. Bevacizumab-resistant colorectal cancer cells and their respective exosomes carry increased levels of the lncRNA SNHG11. SNHG11 increases MRP1 expression via downregulation of miR-1207-5p, resulting in Bevacizumab resistance [228].

Most small molecules used in targeted therapy are protein-kinase inhibitors, thus interfering with cell proliferation signaling. Imatinib-resistant gastrointestinal stromal tumors show an overexpression of Rab25, which leads to the increased release of exosomes. These exosomes have been shown to transfer the resistant phenotype to sensitive cells [229]. Exosomes from Imatinib-resistant chronic myeloid leukemia cells carry elevated levels of IFITM3, CD146, and CD36 and can increase Imatinib tolerance in recipient cells [230]. Exosomes released from lung cancer cells treated with Osimertinib can transfer wildtype EGFR to recipient EGFR-mutated cells, increasing their Osimertinib resistance [231]. Another study found the exosomal transfer of miR-210-3p to be involved in Osimertinib resistance transmission [232]. Exosomal delivery of mir-21 was associated with Gefitinib resistance in lung cancer cells [233]. Another study found that exosomal miR-564 and miR-658 derived from Gefitinib-resistant lung cancer cells can induce resistance in recipient cells [234]. Treatment of melanoma cells with Vemurafenib can enhance miR-211-5p expression in cells and exosomes, allowing for resistance transfer [235]. Another study found circRNA hsa_circ_0001005 to be elevated in Vemurafenib-resistant cells and their respective exosomes. In recipient cells, hsa_circ_0001005 functions as a miRNA sponge, thereby activating canonical pathways related to drug resistance [236].

In summary, more detailed research is needed investigating the effect of exosomes on the outcomes of immune- and targeted therapies. However, given the involvement of exosomes in numerous immunological processes, it is likely that exosomes also contribute to the success or failure of these immunotherapies.

### 7.3. Radiotherapy

Radiotherapy uses ionizing radiation to damage or kill cancer cells locally. Radiation elicits its toxic effects through the direct damage of hit molecules or indirect effects, mostly due to the ionization of the water contained in the cell plasm, which leads to fission products and radicals with oxidative properties. Both direct and indirect effects induce DNA damage and subsequent cell cycle arrest or cell death. Radiotherapy can be applied with curative intent or as adjuvant or palliative treatment. It is often combined with chemotherapy or surgery [237].

As for chemo- and immunotherapy, radiotherapy also increases the general number of exosomes that are secreted. In non-human primates, the number of plasma-derived exosomes per µL was elevated after irradiation [238]. Breast cancer cells have been shown to release increased amounts of exosomes after irradiation in a dose-dependent manner [239]. A possible explanation is the stress-induced activation of p53, which has been proposed as a regulator of exosome biogenesis in a TSAP6-dependent manner [240,241].

There is also evidence suggesting the role of exosomes in the resistance of cancer cells to radiotherapy (Figure 3C). In lung cancer cells, irradiation leads to increased levels of exosomal miR-208a, which decreases apoptosis and increases proliferation in recipient cells, leading to a radioresistant phenotype [242]. Pancreatic cancer cells dying from irradiation release exosomes carrying miR-194-5p, which induces G1/S arrest and enhances the DNA damage response, promoting the survival of tumor-repopulating cells [243]. Irradiated glioma cells secrete exosomes with elevated levels of miR-889, Notch, and JAK-STAT, favoring the proliferation of recipient cells, while the levels of tumor-suppressive miR-516 and miR-365 are decreased [244]. Exosomal miR-208a from irradiated cells increased the proliferation and radioresistance of lung cancer cells by targeting p21 and the AKT/mTOR pathway [242]. Radiotherapy-resistant colorectal cancer cells and their respective exosomes show elevated levels of miR-19b. Transfer of miR-19b to naïve cells via exosomes leads to a reduction in apoptosis and an increase in 3D spheroid formation ability, indicating radioresistance and stemness properties [245]. The abundance of the circular RNA circMYC was found to be increased in exosomes from nasopharyngeal cancer and could be associated with disease recurrence. In vitro experiments revealed that the overexpression of circMYC increases resistance to radiotherapy and promotes proliferation, while its knockdown has the opposite effects [246]. Low-dose radiotherapy of glioblastoma cells induces high levels of circMETRN in exosomes, which increases the radioresistance of recipient cells via miR-4709-3p/GRB14/PDGFRα signaling [247]. Using HNSCC cells, one study found 472 proteins that were differentially expressed in exosomes after irradiation. These proteins were mainly involved in the radiation response, radical oxygen species metabolism, DNA repair, chromatin packaging, and protein folding [248]. Exosomes from irradiated cervical cancer cells incorporate increased levels of survivin, promoting recurrence after therapy [249]. Exosomes from LMP1-expressing nasopharyngeal cancer cells can transfer a resistant phenotype to recipient cells via P38 MAPK signaling [250]. In an HNSCC cell model, exosomes from irradiated cells increased DNA double-strand break repair in recipient cells, leading to a higher irradiation tolerance [251]. In rectal cancer, exosomal proteins C8G, CFHR3, and SerpinF2 were elevated in patients with a good response to radiotherapy. The same study found that the exosomal metabolite 1,4-Dithiothreitol is elevated in poor responders, proposing exosomes as possible biomarkers for radiotherapy efficiency in rectal cancer [252]. In human and canine breast cancer cell models, radiotherapy-resistant cells could transfer their resistance to naïve cells via exosomes and additionally increase their resistance to chemotherapy with doxorubicin. The same study also showed that pre-incubation with exosomes derived from radiotherapy resistance increases the 3D-spheroid-forming ability of recipient cells [253]. Glioblastoma cells treated with exosomes from irradiated cells show increased motility [254]. This could explain the local and distant spreading of tumors often observed after radiotherapy [255]. Another major hurdle in radiotherapy is hypoxia, as oxygen is needed to produce free radicals through ionization. Tumors typically contain hypoxic regions distributed throughout the whole tumor mass [256]. As previously mentioned, exosome release is increased under hypoxic conditions [73]. In esophageal SCC cells, hypoxic exosomes were found to be enriched in miR-340-5p, which accelerated DNA damage repair and induced radioresistance in normoxic cells [256].

Another challenge in radiotherapy is the radiotherapy-induced bystander effects (RIBEs), which describe damaging effects on neighboring cells that have not been directly exposed to radiation but received damaging signals through cell–cell communication with affected cells. Exosomes contribute to this intercellular communication and can induce RIBEs. Exosomes from irradiated keratinocytes lead to increased ROS production and cell death in naïve cells [257]. Irradiated fibroblasts release exosomes enriched in miR-21, which induces the formation of micronuclei and enhances DNA damage in bystander cells [258]. Similar effects were observed in keratinocytes [259]. Exosomal miR-1246 from irradiated bronchial epithelial cells also induces micronuclei formation and DNA damage in bystander cells, inhibiting their proliferation [260]. Exosomes from irradiated bronchial epithelial cells can transfer miRNA-7-5p to non-irradiated cells to induce autophagy via the EGFR/Akt/mTOR axis [261]. One study using breast cancer cells showed that even bystander cells that experienced RIBE can transfer the effects further onto naïve cells via exosomes [262].

Radiation therapy can also cause radiation burns. Depending on the dose of radiation, the effects can range from rather benign erythema to chronic non-healing ulcers and fibrosis. A key factor in healing burn wounds is mesenchymal stem cells (MSCs) [263]. MSC-derived exosomes exert immunomodulatory functions, regulating immune cell function and cytokine release [264]. For example, the release of exosomes enriched in miR-146 and miR-34 from MSCs induces M2-like macrophages [265]. MSC-derived exosomes carrying miR-135a promote epithelial cell migration to the burn site [266]. Exosomes from MSCs can also reduce EMT and thus inhibit fibrosis [267].

In conclusion, not only do exosomes contribute to radiotherapy resistance by increasing DNA damage repair and delivering pro-survival signals, counteracting the toxic effects of radiation, but they can also lead to unwanted RIBE. But on the contrary, exosomes derived from MSCs can also facilitate healing and regeneration of radiotherapy-induced burn wounds.

## 8. Conclusions and Future Directions

Exosomes are important intercellular messengers, delivering complex cargoes that alter the recipient cells’ fate. As such, they also have a pivotal impact on cancer development and progression. There is already a large body of evidence suggesting the involvement of exosomes in cancer progression, metastasis, and therapy response, with new mechanisms emerging continuously. Since therapy response and the development of resistance are still major concerns in cancer treatment, discovering the underlying mechanisms is of critical importance. Exosomes have been shown to transfer drug exporters and pro-survival signals from therapy-resistant to sensitive cells, leading to a reduction in apoptosis and increased survival. There is also a vast amount of evidence that exosomal delivery of miRNAs and other ncRNAs is involved in the evasion of apoptosis, resulting in resistance to chemo-, immuno-, and radiotherapies. Regarding the fact that the majority of studies are focusing on RNAs, further research is urgently needed to clarify the potential functions of other exosomal cargoes, such as membrane and soluble proteins, metabolites, and DNA, in therapy resistance. In chemotherapy, the export of the active drug via exosomes also offers a resistance mechanism, while in immunotherapy, exosomal surface proteins can serve as decoys for drugs, decreasing their efficiency. In radiotherapy, exosomes are not only involved in resistance transfer but also contribute to radiotherapy-induced bystander effects (RIBEs), resulting in unwanted adverse effects on surrounding healthy tissue. On the other hand, exosomes have also been implicated in sensitizing cells to chemotherapy and aiding regeneration in radiotherapy-induced burns.

**Figure 4:**
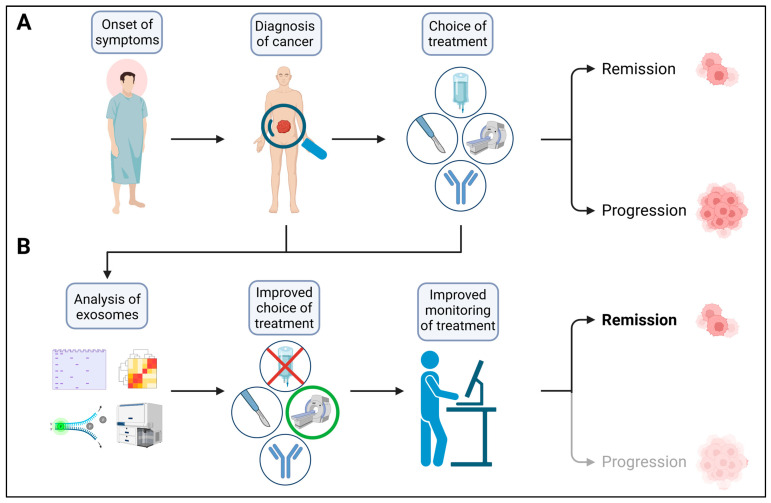
Clinical use of exosomes. (**A**) After the diagnosis of cancer, an appropriate treatment strategy (surgery, chemotherapy, radiotherapy, immunotherapy, or a combination) is chosen based on the clinical gold standard. (**B**) Concurrent and recurrent analyses of exosomes allow treatment choices to be optimized and closely monitored, resulting in higher chances of remission and improved patient survival. Created with BioRender.com.

One of the main goals for future research is to make use of these discoveries to improve the clinical management of cancer patients (Figure 4). While numerous potential applications of exosomes for clinical implementation have already been proposed, including their use as biomarkers for cancer disease in general, as well as for potential drug resistance, as drug carriers, and even as therapy targets, their integration into standard practice is still very limited. This can be attributed to the lack of easy-to-use, standardized isolation and analysis protocols and the rather small amount of in vivo data compared to in vitro studies. Translating the current knowledge about exosomes in cancer progression and therapy response into clinical practice will be the aim of future research. Therefore, there is a high need for (observational) clinical studies evaluating the prognostic power of concurrent exosome analysis during standard cancer treatment (Figure 4B). We suggest that focusing on the development of standardized and potentially automated isolation methods will aid in the successful integration of exosomes as biomarkers into clinical use. Exploiting exosomes as targeted drug carriers is another promising approach, given their high biocompatibility. Further research into the in vivo application of such exosome-based drugs will pave the way for their clinical integration. Analyzing and comparing the biodistribution of heterologous and autologous exosomes will be a pivotal step in this regard. A rather new therapeutic approach is targeting cancer-associated exosomes by inhibiting their release or uptake and, in turn, their cancer- and therapy-resistance-promoting effects. Whether such inhibition can be integrated into clinical practice remains to be elucidated, as it has the potential to interfere with numerous physiological processes in vivo. However, the evidence supporting exosomal involvement in several steps of cancer progression and the transfer of resistant phenotypes renders these vesicles an attractive target to be investigated in depth in future clinical research.

## Data Availability

Not applicable.
